# Protective effect of coenzyme Q10 against doxorubicin-induced cardiotoxicity: Scoping review article

**DOI:** 10.1016/j.jsps.2023.101882

**Published:** 2023-11-21

**Authors:** Al Qahtani Abdullah, Al Balawi Hamed, Al Jowesim Fahad

**Affiliations:** a King Faisal University, Collage of Clinical Pharmacy, Alahsa, Saudi Arabia; b Ministry of National Guards Health Affairs, King Abdulaziz Hospital, Alahsa, Saudi Arabia; c Ministry of Health- Regional poison control center, Dammam, Saudi Arabia

**Keywords:** Coenzyme Q10, Doxorubicin, Cardiotoxicity, Cardioprotective, Ubiquinone

## Abstract

**Introduction:**

Doxorubicin (dox) is classified as an antineoplastic antibiotic which is known as adriamycin from the anthracycline group. Due to the release of free radicals and lipid peroxidation which can cause acute cardiotoxicity. Coenzyme Q10 is found in many cells of the body, it is an antioxidant that reduces oxidative stress and lipid peroxidation.

**Aim:**

This scoping review aims to evaluate the cardioprotective effect of coenzyme Q10 in doxorubicin-induced cardiotoxicity in animals.

**Methods:**

This review was done based on Arksey and O’Malley's methodology, reviewing published articles from October 1978 and September 2023.

**Results:**

14 out of 11,303 articles were included from the initial search, (10 out of 14 articles found that coenzyme Q10 protect has a protection effect against doxorubicin-induced cardiotoxicity).

**Conclusion:**

The results of this review found coenzyme Q10 protects against doxorubicin cardiotoxicity. It is a promising supplement that could be used to prevent cardiotoxicity induced by doxorubicin in cancer patients.

## 1 Introduction

Doxorubicin, classified as an antineoplastic antibiotic and known as Adriamycin, belongs to the anthracycline group. This drug inhibits DNA and RNA synthesis, as well as topoisomerase II activity, and forms an iron chelating complex that binds to cell membranes and DNA (Meredith and Dass, 2016). Doxorubicin is utilized in treating various types of cancer, including both hematological malignancies and solid tumors. Among the side effects associated with doxorubicin are neutropenia, nausea, vomiting, hair loss, and cardiac toxicity. The drug's usage has been constrained by cardiotoxicity linked to cumulative doses. (Chatterjee et al., 2010, Christidi and Brunham, 2021)(See Table 1).

**Table 1:** (Review for doxorubicin and coenzyme Q10 from 1978 to 2023).

**Author**	**Country**	**Main finding**	**Dose/duration (Q10)**	**Dose/duration (doxorubicin)**	**Type of animal**	**Indication of doxorubicin**	**Histology**
Zuowei Pei 2022	China	Cardioprotective	100 mg/kg once a day for 8 weeks	Intraperitoneal injections with DOXORUBICIN (5 mg/kg) once weekly for 4 weeks	C57BL/Mice	Cardiotoxicity induction	Yes
Dalia A. Shabaan 2021	Egypt	Cardioprotective	1 mg/kg/day for 7 days	12.5 mg/kg IP as a single dose	Rats	Cardiotoxicity induction	Yes
Brian Sacks 2021	USA	Cardioprotective	Not clear	Not clear	Rats	Cardiotoxicity induction	Yes
Ana Flavia M. Botelho 2019	Brazil	Cardioprotective	Q10 (1 mg/kg) orally daily	doxorubicin 1 ml of Nacl daily	Rats	Cardiotoxicity induction	Yes
Hesham N. Mustafa(2017)	Egypt	Cardioprotective	1.CoQ10 200 mg/kg 2.L-carnitine 100 mg/kg (started 5 days before dox and continued for 10 days)	Doxorubicin group 10 mg/kg	Female Wistar albino rats	Cardiotoxicity induction	Yes
Pei-Yu Chen 2016	China	Cardioprotective	CoQ10 oral (10 mg/kg in 10 ml (3 weeks)	doxorubicin (2.5 mg/kg, in 2 ml saline)	Rats	Cardiotoxicity induction	Yes
Ofelia Tabora 2009	USA	Not clear	1. Q10 10 mg/kg 2. tocopherol 100 mg/kg	doxorubicin 10 ml/kg (3 days)	Mice	Cardiotoxicity induction	No
Qingyu Zhou and balram Chowbay 2002	Singapore	Not specific on cardiac (Q10 did not increase excretion of doxorubicin)	CoQ10 20 mg/kg for 6 days before doxorubicin	Doxorubicin 10 mg/kg	Rats	Pharmacokinetic study	No
H. Muhammed and C. K. Ramakrishna 1983	India	Not clear	Q10 0.5 mg in 0.2 ml of peanut oil(8 days)	doxorubicin 0.5 mg in 0.2 ml of water daily	Rats	Toxicity induction	No
N. Shimamoto 1982	Japan	Cardioprotective	Q10 3 mg/kg	1.doxorubicin 1 mg/kg 2. QMDP-66 1 mg/kg	Rats(3 days)	Cardiotoxicity induction*	yes
Tadao Usui 1982	Japan	Cardioprotective	after cumulative dose (doxorubicin 1 mg/kg and CoQ10 2.5 mg/kg three times weekly)	1 mg/kg/day ADR i.v. three times a week	Rabbits	Cardiotoxicity induction	Yes
Hideto Ohhara 1981	Japan	Cardioprotective	Q10 15 mg/kg (7 dyas) then	doxorubicin 15 mg/kg	Rats	Cardiotoxicity induction	No
Choe JY 1979 (only abstract)		Cardioprotective	Need full article		Rats	Cardiotoxicity induction	Need full article
Karl Folkers (1978)	Austin, Texas	Insufficient data	CoQ10 at 1 mg/ml per kg was administered.(Monday-Thursday) CoQ10 in the morning and doxorubicin in the afternoon)	adriamycin at 1 mg/2 ml per kg	Male Sprague-Dawley rats	Cardiotoxicity induction	No

*doxorubicin was used to induce cardiotoxicity in healthy animal model.

Cardiotoxicity can arise from the release of free radicals and lipid peroxidation, which may cause acute cardiotoxicity (reversible) that is not dose-related and occurs during or immediately after a single dose of doxorubicin. Alternatively, chronic toxicity (irreversible) is dose-related and characterized by a reduced ejection fraction and symptomatic heart failure, which can occur months to years after the completion of treatment. (Meredith and Dass, 2016, Chatterjee et al., 2010, Christidi and Brunham, 2021).

Coenzyme Q10 (Q10) or ubiquinone is ubiquitous in many cells of the body, such as the lungs, heart, and muscles, and is particularly concentrated in the mitochondria (Niklowitz et al., 2007). It can be utilized as a supplement for patients undergoing chemotherapy and for those with heart diseases, being considered a safe adjunct (Niklowitz et al., 2007). The production of Q10 decreases with age, and supplementation may become necessary for the elderly. It functions as an antioxidant, reducing oxidative stress and lipid peroxidation (Bentinger et al., 2007, Genova et al., 2003). The suggested dose of coenzyme Q10 is that serum targets of > 2 mg/L are the levels to achieve clinical outcomes, after oral Q10 administration. However, Q10 has low oral bioavailability, which limits its cardioprotective use; an alternative method to increase bioavailability is the application of a chitosan coating (Quagliariello et al., 2020). The utility of doxorubicin (dox) is constrained due to cardiotoxicity resulting from cumulative dosing. This review aims to evaluate the cardioprotective effects of coenzyme Q10 on doxorubicin-induced cardiotoxicity in animal models.

## 2 Methods

This review used the five-step methodology of Arksey and O’Malley (2007) (Özsu, 2009). The guide proposed by Levac et al. (2010) was used in operationalizing each step. (Hacking, 2012).

Step 1: Identification of the research question.

This article was conducted to map evidence on the cardioprotective effect of coenzyme q10 against Doxorubicin-induced cardiotoxicity.

A preliminary search using the terms “coenzyme Q10“ and doxorubicin”.

A large number of articles. Most articles were not related to our research question.

Therefore, we proceeded to identify relevant articles by adding “cardiac toxicity” to search terms.

The specified search terms were “coenzyme q10 and doxorubicin cardiac toxicity” We identified one broad research question as follows:

What is known, from existing literature for the period?

From October 1978–2023- about the efficacy of coenzyme q10 as cardioprotective agents against doxorubicin-induced cardiotoxicity in animals?

Step 2: Identification of relevant studies.

Before the literature search, the inclusion and exclusion criteria were determined.

The inclusion criteria were used to guide the search and were as follows:

• **Study type:**
1- Preclinical studies.
• **Publication:**
1- Fully published article.
2- Abstracts
• **Population**:

1. Mouse.

2. Rabbits.

3. Animal organs (rat heart).

4. Rats.

• **Comparison**:

Any comparison.

• **Outcome:**

Cardioprotective properties.

• **Language:**

Articles in English.

• **Publication period:**

Publications from October 1978- September 2023.

• **The exclusion criteria included:**

Studies were published in a language other than English, as English was the common denominator language for all the authors.

1- Systemic review.
2- Studies done on humans.
3- Studies not related to the research question.
4- Toxicity induced by doxorubicin is not related to the heart.
5- Studies used combination with coenzyme Q10.
• **Search strategies**

Publication titles from the preliminary search were reviewed and used to refine the terms for this review. The search was conducted using, Scopus, Web of Science, Science Direct, PubMed, Google Scholar, EMBASE, and Cochrane database.

• Keywords **and terms:**

Keywords including: “coenzyme q10”, “doxorubicin”, and “cardiac toxicity” were used in the search terms;

“coenzyme q10 and doxorubicin cardiac toxicity” and “coenzyme q10 and doxorubicin cardioprotective” were also used.

Step 3: Study selection.

After an extensive search, inclusion, and exclusion criteria.

**Chart 1:**
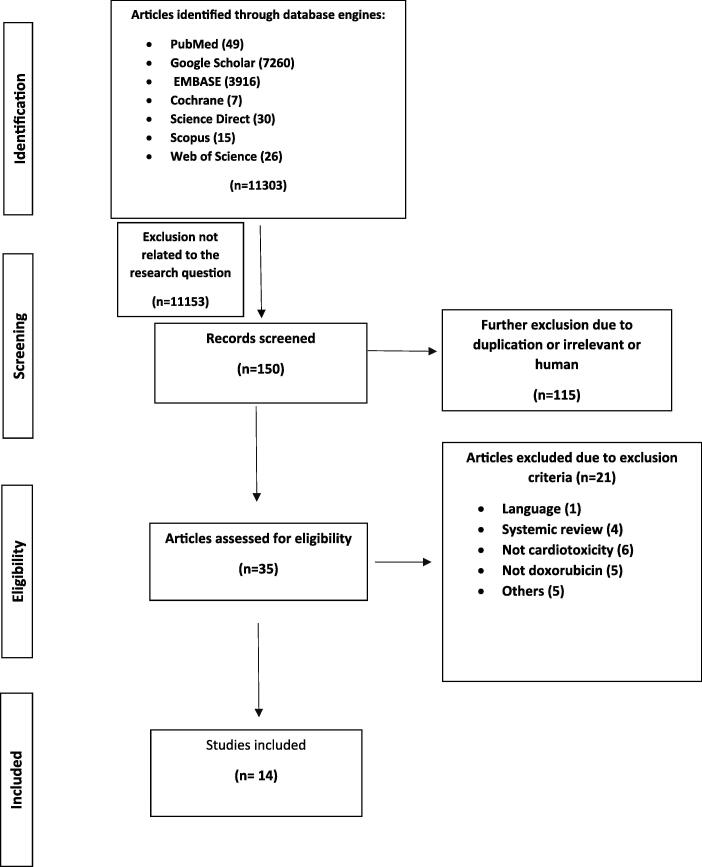

Were independently applied by three reviewers to all the citations. The reviewers aimed to select the articles that matched the research question. We justified the exclusion of the studies from this review (Chart 1).

Step 4: Charting the data.

A data-charting form was developed and agreed upon by the reviewers; this was used to extract data from the literature. Information from eligible articles was extracted by the first author and reviewed by the other authors (Chart 1).

Step 5: Collating, summarizing, and reporting results.

The data was captured and summarized in an Excel sheet.

## 3 Results

Different doses and duration of coenzyme Q10 have been used to assess cardio-protection against doxorubicin cardiotoxicity. The included trials out of 11,303 were 14 articles, out of the 14 trials 10 of the articles (71.4 %) concluded that coenzyme Q10 protects against doxorubicin cardiotoxicity (Pei et al., 2022, Shabaan and Mostafa, 2021, Sacks et al., 2021, Botelho et al., 2020, Mustafa et al., 2017, Pei-Yu Chen et al., n.d., Shimamoto et al., 1983, Usui et al., 1982, Ohhara et al., 1981, Choe et al., 1979). Two of the 14 (14.2 %) articles focus on survival and oxygen uptake and their conclusion was not clear and needs further investigation (Aldridge, 1960, Tábora et al., 1986). One of the articles (7.1 %) focuses on the effect of coenzyme Q10 on the pharmacokinetics of doxorubicin, the results showed that coenzyme Q10 will not increase the excretion of doxorubicin and it will not affect its cytotoxicity on cancer cells (Zhou and Chowbay, 2002). Two of the articles (14.2 %) had insufficient data to conclude that coenzyme Q10 can protect against doxorubicin cardiac toxicity, The Second study that had insufficient data focused on change in QRS and concluded that coenzyme Q10 may decrease some of the toxicity of doxorubicin (Folkers et al., 1978). Two of the articles (14.2 %) compared coenzyme Q10 with other agents, first study coenzyme Q10 compared with L-carnitine and it is the only article measured cardiac biomarkers in addition to oxidative status and conclude that coenzyme Q10 or L-carnitine can protect against doxorubicin cardiotoxicity (Mustafa et al., 2017). Second article coenzyme Q10 compared with QMDP-66 which focuses on heart rate and ECG changes concludes that coenzyme Q10 or QMDP-66 can protect against doxorubicin cardiotoxicity. (Shimamoto et al., 1983).

## 4 Discussion

In this review, the aim was to evaluate the possible cardio-protective effects of coenzyme Q10 against cardiotoxicity induced by doxorubicin in pre-clinical studies.

This review found that 10 out of 14 of the pre-clinical studies included support that coenzyme Q10 has a protective effect against doxorubicin cardiotoxicity induced on the experimented animals or organs.

The cardiotoxicity induced by doxorubicin remains ambiguous and not fully understood (Coull and Wellenius, 2012), but the generation of reactive oxygen species (ROS) caused by Doxorubicin is a primary mechanism that leads to myocardium injuries, it also induces lipid peroxidation and apoptosis (Oktem et al., 2012). One study says that cardiotoxicity induced by doxorubicin is a result of the expression of the β‐myosin heavy chain isoform with a trend to increase atrial natriuretic factor and the activation of p38 MAPK, which is the leading cause of contractile dysfunction and atrophy (McLean et al., 2019). Another study said that doxorubicin significantly decreased Nrf2 content in heart tissues at the same time it increased the generation of ROS and free radicals in cardiomyocytes, which leads to myocardial function impairment. Kabel et al., 2021, Rahmanifard et al., 2021.

In the metabolism of doxorubicin, the main pathway is the reduction of carbonyl-yielding cytotoxic doxorubicinol. doxorubicinol is a cytotoxic metabolite circulating in treated patients and animals (Behnia and Boroujerdi, 2010, Takanashi and Bachur, 1976), it has been found to inhibit ATPases of mitochondrial membrane, sarcolemma, or sarcoplasmic reticulum from rabbit or dog hearts. (Boucek et al., 1987).

Doxorubicinol is a metabolite of doxorubicin, and it alters iron hemostasis in the body, this is on the other hand a reason for life-threatening cardiomyopathy induced by doxorubicin (Boucek et al., 1987, Olson et al., 1988). Furthermore, doxorubicin reduces the mitochondrial coenzyme Q10 which has a protective effect on the heart from injuries. (Pei-Yu Chen et al., n.d., Zeisel, 2004).

Co-enzyme Q10 as a powerful antioxidant showed cardioprotective properties through many mechanisms, it preserved myocardial ATP levels (Muller, 2017), it also showed a reduction of ROS production and increased the superoxide dismutase and glutathione peroxide activity, and it also improved the malondialdehyde (MDA) concentration and the activity of catalase and mitigate their changes (Rahmanifard et al., 2021). Co-enzyme Q10 decreases the doxorubicinol and somehow increases the production of doxorubicinolone, but linking this effect to its cardiac protection is unproved (Zhou and Chowbay, 2002). Antioxidant effect of coenzyme Q10 takes place by scavenging free radicals or regenerates tocopherol and ascorbate from an oxidized state (Tsuneki et al., 2007). ECG, heart weight, and heart structure were changed on rats treated with doxorubicin.

On the other hand, coenzyme Q10 gave protection against ECG changes and reduced heart remodeling. Heart weight increased with doxorubicin, while weight reduction of the heart was seen with coenzyme Q10 administration with doxorubicin. (Mustafa et al., 2017).

Histopathological findings indicate that doxorubicin causes apoptosis and fibrosis in the heart. However, with the administration of coenzyme Q10, inflammatory cell infiltration in the myocardium, myofibril disorganization, and exudation have been decreased (Mustafa et al., 2017). Transformation of fibroblasts to myofibroblasts, which leads to the production of collagen and finally cardiac fibrosis, was also reduced by coenzyme Q10. (Mustafa et al., 2017, Songbo et al., December 2018).

Due to the cardiotoxicity of doxorubicin, a cumulative dose should not be exceeded in the lifetime of the patients (Meredith and Dass, 2016, Chatterjee et al., 2010, Robert et al., 2011). In this review, the coenzyme Q10 showed a cardioprotective effect but the limitation of the lifetime dose was not proven to be increased or affected by coenzyme Q10 protection properties. This directs us to another question that needs to be investigated, whether coenzyme Q10 can increase the range of doxorubicin cumulative dose or not. Further studies are required.

Previous studies indicate that the cytotoxicity of doxorubicin on cancer cells was not affected by the administration of coenzyme Q10 (Greenlee et al., 2012). This is an advantage supporting supplementation of coenzyme Q10 concurrently with doxorubicin. Therefore coenzyme Q10 is a strong candidate for supplementation with doxorubicin and to be evaluated in clinical trials to test its cardioprotective effect on humans.

Some preclinical studies suggest using coenzyme Q10 as an adjuvant with doxorubicin-treated patients due to its cardioprotective effects (Rahmanifard et al., 2021, Sundukov, 2006), another preclinical study suggests using coenzyme Q10 pre and post-doxorubicin treatment proved cardioprotective effects. (Botelho et al., 2020).

The antioxidant effect of coenzyme Q10 and its cardioprotective properties pre-clinically has been proven in most of the studies in this review. It seems that coenzyme Q10 is a promising antioxidant that could protect against the cardiotoxicity induced by doxorubicin, thus human trials need to be done to investigate its effect on cancer patients having doxorubicin in their regimen.

## 5 Conclusion

The result of this review indicates the potential cardioprotective effects of coenzyme Q10 against doxorubicin cardiotoxicity. Coenzyme Q10 reduces oxidative stress, myocardium injuries, and ECG and structural changes in the heart. Even with antagonizing the cardiotoxicity of doxorubicin by coenzyme Q10, the anti-tumor effect was not demolished. Coenzyme Q10 seems to be a promising supplement protecting from cardiotoxicity in patients treated with doxorubicin.

## Declaration of competing interest

The authors declare that they have no known competing financial interests or personal relationships that could have appeared to influence the work reported in this paper.
